# FunctanSNP: an R package for functional analysis of dense SNP data (with interactions)

**DOI:** 10.1093/bioinformatics/btad741

**Published:** 2023-12-07

**Authors:** Rui Ren, Kuangnan Fang, Qingzhao Zhang, Shuangge Ma

**Affiliations:** Department of Biostatistics, Yale School of Public Health, New Haven, CT 06520, United States; Department of Statistics and Data Science, Xiamen University, Xiamen 361005, China; Department of Statistics and Data Science, Xiamen University, Xiamen 361005, China; The Wang Yanan Institute for Studies in Economics, Xiamen University, Xiamen 361005, China; Department of Biostatistics, Yale School of Public Health, New Haven, CT 06520, United States

## Abstract

**Summary:**

Densely measured SNP data are routinely analyzed but face challenges due to its high dimensionality, especially when gene–environment interactions are incorporated. In recent literature, a functional analysis strategy has been developed, which treats dense SNP measurements as a realization of a genetic function and can ‘bypass’ the dimensionality challenge. However, there is a lack of portable and friendly software, which hinders practical utilization of these functional methods. We fill this knowledge gap and develop the R package FunctanSNP. This comprehensive package encompasses estimation, identification, and visualization tools and has undergone extensive testing using both simulated and real data, confirming its reliability. FunctanSNP can serve as a convenient and reliable tool for analyzing SNP and other densely measured data.

**Availability and implementation:**

The package is available at https://CRAN.R-project.org/package=FunctanSNP.

## 1 Introduction

Densely measured SNP data are now routinely analyzed. However, high data dimensionality often poses serious challenges to analysis, leading to inferior computational and numerical performance. These challenges may exacerbate when gene–environment interactions are incorporated (Liu *et al.* 2013, Manuck and McCaffery 2014). Multiple strategies have been proposed. In recent studies (Fan *et al.* 2013, Zhang *et al.* 2016), a functional data analysis strategy has emerged and demonstrated high effectiveness. It has been motivated by the observation that SNPs that are physically close tend to be in linkage disequilibrium and have similar behaviors. Here, the densely measured discrete SNPs are viewed as a realization of an underlying genetic function. In practice, such functions can be effectively approximated using a much smaller number of basic functions, hence significantly reducing dimensionality. Additionally, this functional strategy may also improve the stability and interpretability of estimation (Ramsay and Silverman 1996). Multiple methods have been developed for the analysis of main effects only as well as with interactions. We refer to the original publications and Supplementary Materials for more information. However, there is a lack of portable and friendly software implementing such methods, which hinders utilization in daily practice. Our goal is to develop an R package and fill this knowledge gap.

## 2 Methods

**Figure 1.**
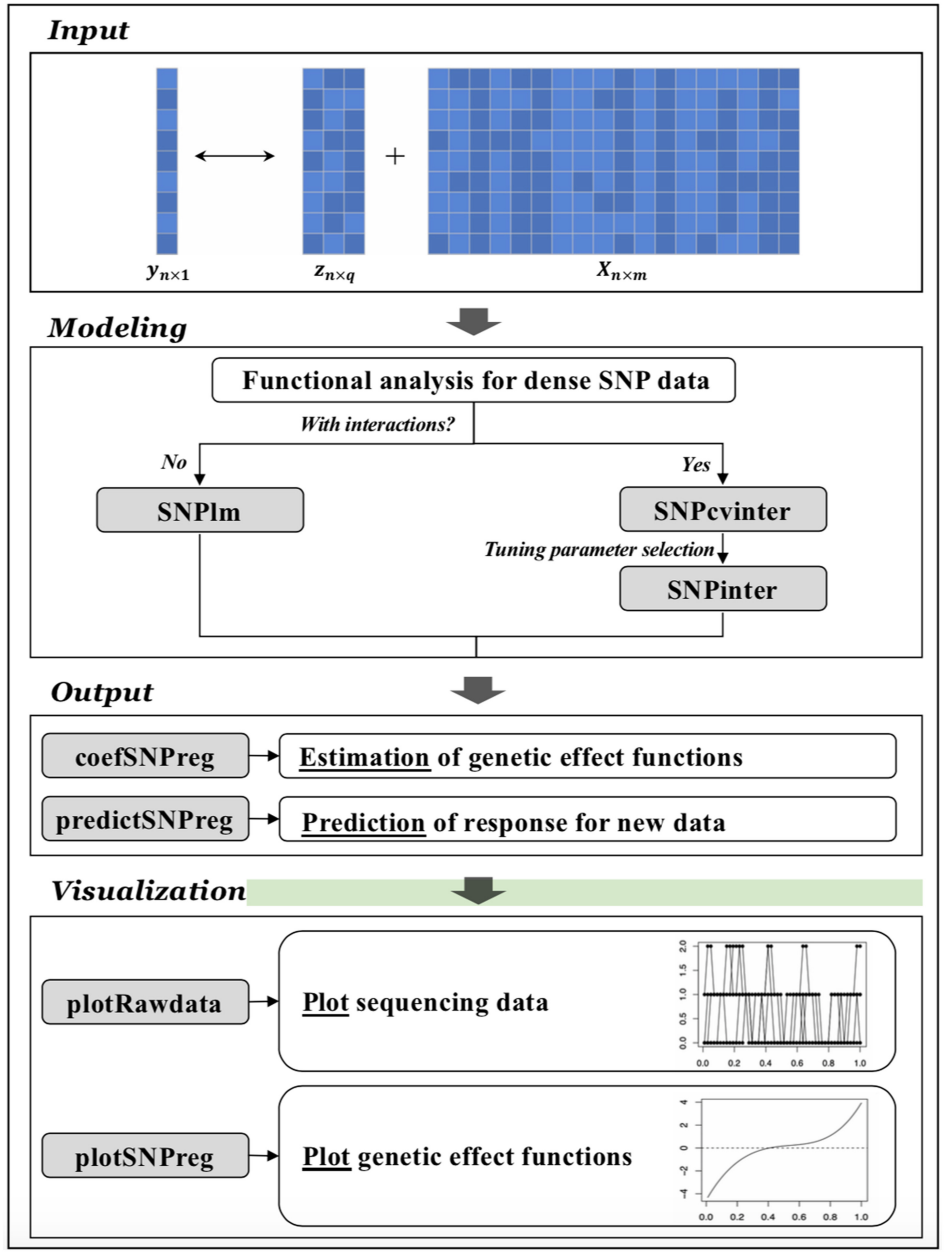
Main workflow.

The FunctanSNP package has been published at the Comprehensive R Archive Network (CRAN). Its main workflow is presented in Fig. 1. It is designed to quantify the association between a quantitative trait and densely measured genetic variants (that have physical location information available) and scalar covariates. The package conducts two main sets of analysis, as detailed in the Supplementary Materials.

- SNPlm. This model was first developed by Fan *et al.* (2013). It involves a regression model incorporating the additive effects of scalar covariates and genetic variants. Employing functional modeling techniques, it treats both genetic variants and their effects as functions of the variants’ locations. With the popular basis expansion technique, a least squares estimation is conducted.
- SNPinter. In the literature, there has been limited research on functional interaction models for genetic variation analysis (Lin *et al.* 2017, Liang *et al.* 2023). This model adopts a regression setup with the additive effects of scalar covariates, genetic variants, and their interactions. Genetic variants are modeled using functional data analysis techniques. Their main and interaction effects are also assumed to be functions of the variants’ locations. With the consideration that not all regions of genetic variants are relevant, a functional sparse group Minimax Concave Penalty technique is applied to identify relevant genetic regions and respect the “main effects, interactions” hierarchy. It is noted that this technique has been only recently developed and is nontrivial. The methodological and computational details are available in the Supplementary Materials.

As described in the Supplementary Materials, the package flexibly allows setting user-specified tunings and using cross-validation to select optimal tunings. It then performs estimation under the selected tunings and provides graphical and numerical output. In the Supplementary Materials, we provide demo codes and corresponding sample output.

Additionally, to “validate” the software functions and get a better understanding of the analysis, in the Supplementary Materials, we conduct simulation and comparison with competing alternatives. The accurate and superior estimation and selection performance can provide strong support to the software and promote practical utilization.

## 3 Application notes

A detailed user manual has been developed and is available at CRAN, which facilitates installation and utilization. We use the SNPinter analysis as an example. With fixed tunings, it is realized using:

SNPinter(y,z,location,X,lambda1,lambda2,eta,

type1="Bspline",nbasis1=5,params1=4,

Bsplines=71,norder=4,intercept=FALSE,

eps=1e-2,maxstep=1e2,Plot=FALSE)

where X and location are the genetic variants and corresponding locations, y is the response matrix, z contains the covariates, lambda1, lambda2, and eta are the selected tuning parameters, type1, nbasis1, and params1 are the parameters for basis expansion of the genetic variant function, Bsplines and norder are the parameters for B-spline expansion of the genetic effect function, intercept indicates whether an intercept is included, eps and maxstep are the threshold and the number of iterations that determine whether the algorithm stops, and Plot indicates whether to display the estimation results.

The analysis and software are found to be computationally feasible. For example, with a simulated data (with 200 samples, 2 scalar variables, and 100 SNPs—detailed in Case I, Section 3 of the Supplementary Materials), the analysis with fixed tunings takes about 0.50 s on a desktop with standard configurations, and convergence is achieved within a few iterations. We further conduct the same simulation but with 20 000 SNPs, and the analysis takes about 1.74 s. The much slower than linear increase in computational cost can further justify the merit of the functional-based analysis and suggest the potential applicability of the analysis and software to large-scale data.

To further demonstrate the utility of the analysis and software, we analyze data from the Childhood Asthma Management Program study. The dataset contains 242 samples. For response, we consider forced expiratory volume in 1 s (prebronchodilator) at follow-up, which is an important biomarker for lung function. Two scalar variables, age and gender, are considered. A total of 21 985 densely measured SNPs are analyzed. The estimated functional main and interaction effects for the genetic variants are provided in the Supplementary Materials, where we clearly observe sparsity and the “main effects, interactions” hierarchy. We also resort to a random splitting-based evaluation and observe competitive prediction performance.

## 4 Discussion

We have developed FunctanSNP, the first portable and friendly package that takes a functional perspective and analyzes densely measured SNP data (without and with interactions) along with scalar covariates. It requires basic R settings, can be easily installed and utilized, and exhibits satisfactory performance. Beyond SNP data, it is also applicable to other densely measured data types and can be extended to other types of outcomes and models.

## Supplementary Material

btad741_Supplementary_Data
