# Supplementary material for: FunctanSNP: an R package for functional analysis of dense SNP data (with interactions)
Source: Bioinformatics. 2023 Dec 7;39(12):btad741. doi: 10.1093/bioinformatics/btad741 (PMC10723032; doi:10.1093/bioinformatics/btad741)
Supplement: btad741_Supplementary_Data [file btad741_supplementary_data.pdf]

Supplemental Materials for "FunctanSNP: an R package for functional analysis of dense SNP data (with interactions)" by Rui Ren, Kuangnan Fang, Qingzhao Zhang and Shuangge Ma

This file contains additional details on the methods, software functions, simulation studies, and an application example.

1 Methods

To study the associations between quantitative traits and dense molecular measurements in particular SNPs, we adopt techniques from functional data analysis. We comprehensively consider both partially functional linear and interaction analysis models. It is noted that with minor modifications, the analysis techniques and software can also be applied to other dense/functional variables. The partially functional linear model, referred to as SNPlm, was first proposed in Fan et al. (2013) for testing the associations between quantitative traits and genetic variants. SNPlm treats densely measured genetic variants as realizations of smooth stochastic functions, and then genetic variant functions (GVFs) are estimated. An association analysis is then conducted on the GVFs. Interaction analysis has been routinely conducted with SNPs and other molecular data. We advance the SNPlm model and introduce a partially functional interaction model with local sparsity, named SNPinter. This model allows us to take advantage of SNPlm and can effectively "bypass" the curse of dimensionality of parametric interaction models. A challenge here is the "main effect, interaction" selection hierarchy, which postulates that, if an interaction effect is selected as important, the corresponding main genetic effect also needs to be selected. This hierarchy has been well studied with parametric modeling. However, its functional counterpart has been very limitedly examined.

1.1 SNPgvf

SNPgvf conducts the ordinary least squares smoothing analysis and models the genetic variants of an individual as a genetic variant function.

Consider a dataset with n independent individuals, and individual i is sequenced in a genetic region containing m_i variants. Assume that the m_i variants have been ordered according to their physical locations $t_{i1} < \ldots < t_{im_i}$. To simplify notation, we normalize the region $[\min_{1\le i\le n}\{t_{i1}\}, \max_{1\le i\le n}\{t_{im_i}\}]$ to $[0,T]$. Denote the genotypes of the m_i variants as $G_i = (g_i(t_{i1}), \ldots, g_i(t_{im_i}))^\top$, where $g_i(t_{ij}) \in \{0,1,2\}$ represents the number of minor alleles of the i-th individual and the j-th variant located at t_{ij}, $j = 1, \ldots, m_i$.

With functional data modeling, the genotypes $G_i = (g_i(t_{i1}), \ldots, g_i(t_{im_i}))^\top$ are modeled as a discrete realization of a genetic variant function, denoted as $X_i(t)$, $t \in [0,T]$. Three discrete realization patterns for $X_i(t)$ and G_i have been considered (Fan et al., 2013):

1. Additive effects of minor alleles: $X_i(t_{ij}) = g_i(t_{ij})$.
2. Dominant effects of minor alleles: $X_i(t_{ij}) = 1$ when $g_i(t_{ij}) = 1, 2$, and $X_i(t_{ij}) = 0$ when $g_i(t_{ij}) = 0$.
3. Recessive effects of minor alleles: $X_i(t_{ij}) = 1$ when $g_i(t_{ij}) = 2$, and $X_i(t_{ij}) = 0$ when $g_i(t_{ij}) = 0, 1$.

To estimate $X_i(t)$ using the discrete realizations $\widetilde{\boldsymbol{X}}_i = (X_i(t_{i1}), \ldots, X_i(t_{im_i}))^\top$, we adopt least squares smoothing with a suitable set of basis functions, such as B-spline, Fourier, and wavelet functions (Wang et al., 2016). Specifically, with a set of basis functions $\phi_l(t)$, $l = 1, \ldots, L_X$, we generate the $m_i \times L_X$ matrix $\boldsymbol{\Omega}_i$, which contains the values of $\phi_l(t_{ij})$, $j = 1, \ldots, m_i$, $l = 1, \ldots, L_X$. Subsequently, $X_i(t)$ can be approximated as:

$$\widehat{X}_i(t) = \widetilde{\boldsymbol{X}}_i^\top \boldsymbol{\Omega}_i \left(\boldsymbol{\Omega}_i^\top \boldsymbol{\Omega}_i\right)^{-1} \boldsymbol{\phi}(t), \tag{1}$$

where $\boldsymbol{\phi}(t) = (\phi_1(t), \ldots, \phi_{L_X}(t))^\top$. According to the three discrete realization patterns, the estimated GVFs are classified as additive, dominant, and recessive.

1.2 SNPlm

This model adopts the aforementioned functional modeling strategy. It can accommodate low-dimensional covariate effects when associating genetic variations with a quantitative

trait. For subject $i(=1,\dots,n)$, let y_i denote the quantitative trait and $\boldsymbol{Z}_i = (z_{i1},\dots,z_{iq})^\top$ denote the covariates. Consider the following functional linear model:

$$y_i = \alpha + \sum_{k=1}^{q} z_{ik}\gamma_k + \int_0^T X_i(t)\beta(t)\mathrm{d}t + \varepsilon_i, \tag{2}$$

where α is the intercept, $\boldsymbol{\gamma} = (\gamma_1,\dots,\gamma_q)^\top$ is a $q \times 1$ vector of regression coefficients, $\beta(t)$ is a coefficient function describing the association between the genetic variants and trait, and ε_i is an error term following a normal distribution with mean zero and finite variance σ_e^2.

For $\beta(t)$, we also adopt the basis expansion technique. With a set of basis functions $\boldsymbol{\psi}(t) = \left(\psi_1(t),\dots,\psi_{L_\beta}(t)\right)^\top$ defined in the domain $[0,T]$,

$$\beta(t) = \boldsymbol{\psi}(t)^\top \boldsymbol{b},$$

where $\boldsymbol{b} = \left(b_1,\dots,b_{L_\beta}\right)^\top$ are the unknown basis coefficients. Note that the basis functions used for $\beta(t)$ can be different from those used for $X_i(t)$. With the basis expansions, SNPlm can be rewritten as:

$$\begin{aligned} y_i &= \alpha + \sum_{k=1}^{q} z_{ik}\gamma_k + \left[\widetilde{\boldsymbol{X}}_i^\top \boldsymbol{\Omega}_i \left(\boldsymbol{\Omega}_i^\top \boldsymbol{\Omega}_i\right)^{-1} \int_0^T \boldsymbol{\phi}(t)\boldsymbol{\psi}(t)^\top \mathrm{d}t\right] \boldsymbol{b} + \varepsilon_i, \\ &= \alpha + \boldsymbol{Z}_i^\top \boldsymbol{\gamma} + \boldsymbol{H}_i^\top \boldsymbol{b} + \varepsilon_i, \end{aligned} \tag{3}$$

where $\boldsymbol{Z}_i = (z_{i1},\dots,z_{iq})^\top$ and $\boldsymbol{H}_i^\top = \widetilde{\boldsymbol{X}}_i^\top \boldsymbol{\Omega}_i \left(\boldsymbol{\Omega}_i^\top \boldsymbol{\Omega}_i\right)^{-1} \int_0^T \boldsymbol{\phi}(t)\boldsymbol{\psi}(t)^\top \mathrm{d}t$. Now, SNPlm can be effectively viewed as a "traditional" linear model, with $(\boldsymbol{Z}_i, \boldsymbol{H}_i)$ as the covariates and $(\boldsymbol{\gamma}, \boldsymbol{b})$ as the coefficient vectors. We can obtain the estimate $(\widehat{\boldsymbol{\gamma}}, \widehat{\boldsymbol{b}})$ via the standard linear regression techniques and then estimate the genetic effect function by $\widehat{\beta}(t) = \boldsymbol{\psi}(t)^\top \widehat{\boldsymbol{b}}$.

1.3 SNPinter

With SNPinter, we advance from the above model and accommodate the interactions between the genetic variant function $X_i(t)$ and covariates $\boldsymbol{Z}_i$. This is the functional counterpart of the genetic interaction model – in particular including the gene-environment interaction model as a special case, which has been routinely considered.

1.3.1 Modeling and estimation

Consider the model:

$$y_i = \alpha + \sum_{k=1}^{q} z_{ik}\gamma_k + \int_0^T X_i(t)\beta_0(t)dt + \sum_{k=1}^{q} z_{ik} \int_0^T X_i(t)\beta_k(t)dt + \varepsilon_i. \tag{4}$$

Here, $\beta_0(t)$ and $\beta_k(t)$ are the coefficient functions, where $k = 1, \ldots, q$. The $\beta_k(t)$ functions capture the interactions between $\boldsymbol{Z}_i$ and $X_i(t)$. The error terms ε_i's are assumed to be independent of both $\boldsymbol{Z}_i$ and $X_i(t)$.

In genetic data analysis, sparsity is usually assumed, under which only a subset of genetic variants is relevant for a specific trait. Here, we consider its functional counterpart and adopt a locally sparse estimation technique for $\beta_0(t)$ and $\beta_k(t)$'s. Under this estimation, the functions are exactly zero on certain subregions (which are unknown a priori). Accordingly, the corresponding genetic variants have no contribution to the response. To make the results more interpretable, we consider the functional counterpart of the "main effect, interaction" variable selection hierarchy. In particular, if a subregion has $\beta_0(t) = 0$, then all corresponding $\beta_k(t)$ functions for $k = 1, \ldots, q$ are zero. In our analysis, we consider the common scenario where the covariates are manually selected and likely to be important. As such, sparsity in γ_k's is not considered – it can be easily added if needed.

For estimation, determination of the local sparsity structure, and respect of the variable selection hierarchy, we consider the following objective function:

$$\begin{aligned} Q(\boldsymbol{\beta}(t), \boldsymbol{\gamma}) =& \frac{1}{2n} \sum_{i=1}^{n} \left(y_i - \alpha - \sum_{k=1}^{q} z_{ik}\gamma_k - \int_0^T X_i(t)\beta_0(t)dt - \sum_{k=1}^{q} z_{ik} \int_0^T X_i(t)\beta_k(t)dt \right)^2 \\ &+ \sum_{k=1}^{q} \frac{\kappa}{T} \int_0^T p_{\lambda_1}\left(|\beta_k(t)|\right) dt + \frac{\kappa}{T} \int_0^T p_{\lambda_2}\left(\|\boldsymbol{\beta}(t)\|_2\right) dt + \eta \sum_{k=0}^{q} \int_0^T \beta_k''^2(t)dt, \end{aligned} \tag{5}$$

where $\|\boldsymbol{\beta}(t)\|_2 = \left(\sum_{k=0}^q \beta_k^2(t)\right)^{1/2}$, $p_{\lambda_j}(\cdot)$'s are penalty functions with tuning parameters λ_j's (for $j = 1, 2$), κ is a modifier and will be discussed below, η is a tuning parameter, and $\beta_k''(t)$ is the second order derivative of $\beta_k(t)$ with respect to t. In the numerical study, Minimax concave penalty (MCP) is adopted for both $p_{\lambda_1}(\cdot)$ and $p_{\lambda_2}(\cdot)$. It is defined as $p_{\lambda_j}(t) = \lambda_j \int_0^{|t|} \left(1 - x/\lambda_j\xi\right)_+ dt$, with $\lambda_j \geq 0$ as a tuning parameter and $\xi > 0$ as a regularization parameter (Zhang, 2010). In the context of locally sparse estimation in functional analysis, penalties similar to $\int_0^T p_{\lambda_1}\left(|\beta_k(t)|\right) dt$ have been proposed (Lin et al., 2017). To respect the hierarchy, we treat $(\beta_0(t), \beta_1(t), \ldots, \beta_q(t))$ as a "group". The term $\frac{\kappa}{T} \int_0^T p_{\lambda_2}\left(\|\beta(t)\|_2\right) dt$ determines whether this group of functions has an overall zero effect for a subregion. If not, $\sum_{k=1}^q \frac{\kappa}{T} \int_0^T p_{\lambda_1}\left(|\beta_k(t)|\right) dt$ determines whether (some of) the q interaction effects are nonzero. Note that no penalty is applied directly to $\beta_0(t)$, ensuring that the corresponding estimate

remains nonzero and the hierarchy is respected.

To simplify computation, we adopt the B-spline expansion technique and approximate $\beta_k(t)$ using a set of B-spline basis functions $\boldsymbol{\psi}(t) = \left(\psi_1(t), \ldots, \psi_{L_\beta}(t)\right)^\top$ of degree d. These basis functions are defined over M_n equally-spaced knots in the interval $[0, T]$, where $L_\beta = M_n + d$. The approximation is:

$$\beta_k(t) = \boldsymbol{\psi}(t)^\top \boldsymbol{\theta}_k, \text{ for } k = 0, 1, \ldots, q.$$

Here, $\boldsymbol{\theta}_k$ contains the coefficients for the B-spline basis functions. Denote $\boldsymbol{\theta}$ as a vector that contains all $\boldsymbol{\theta}_k$'s. We set the modifier in (5) as $\kappa = M_n$, which leads to the approximation:

$$\sum_{k=1}^{q} \frac{M_n}{T} \int_0^T p_{\lambda_1}\left(|\beta_k(t)|\right) dt + \frac{M_n}{T} \int_0^T p_{\lambda_2}\left(\|\boldsymbol{\beta}(t)\|_2\right) dt \approx \sum_{k=1}^{q} \sum_{l=1}^{M_n} p_{\lambda_1}\left(\|\boldsymbol{\theta}_k\|_{W_l}\right) + \sum_{l=1}^{M_n} p_{\lambda_2}\left(\|\boldsymbol{\theta}\|_{\boldsymbol{W}_l}\right),$$

where $\boldsymbol{W}_l$ is an $L_\beta \times L_\beta$ matrix with the (i,j)th entry $w_{lij} = \frac{M_n}{T} \int_{t_{l-1}}^{t_l} \psi_i(t)\psi_j(t)dt$ if $l \leq i, j \leq l + d$, and $w_{lij} = 0$ otherwise. $\|\boldsymbol{\theta}_k\|_{\boldsymbol{W}_l} = \left(\boldsymbol{\theta}_k^\top \boldsymbol{W}_l \boldsymbol{\theta}_k\right)^{1/2}$, $\|\boldsymbol{\theta}\|_{W_l} = \left(\sum_{k=0}^{q} \boldsymbol{\theta}_k^\top \boldsymbol{W}_l \boldsymbol{\theta}_k\right)^{1/2}$. Let $\boldsymbol{V}$ be an $L_\beta \times L_\beta$ matrix with the (i,j)th entry $v_{ij} = \int_0^T \frac{d^2\psi_i(t)}{dt^2} \frac{d^2\psi_j(t)}{dt^2} dt$. Then we have:

$$\sum_{k=0}^{q} \int_0^T \beta_k''^2(t) dt = \sum_{k=0}^{q} \boldsymbol{\theta}_k^\top \boldsymbol{V} \boldsymbol{\theta}_k.$$

With this, we can rewrite (5) as:

$$\begin{aligned} Q(\boldsymbol{\theta}, \alpha, \boldsymbol{\gamma}) =& \frac{1}{2n} \sum_{i=1}^{n} \left(y_i - \alpha - \sum_{k=1}^{q} z_{ik}\gamma_k - \int_0^T X_i(t)\beta_0(t)dt - \sum_{k=1}^{q} z_{ik} \int_0^T X_i(t)\beta_k(t)dt \right)^2 \\ &+ \sum_{k=1}^{q} \sum_{l=1}^{M_n} p_{\lambda_1}\left(\|\boldsymbol{\theta}_k\|_{\boldsymbol{W}_l}\right) + \sum_{l=1}^{M_n} p_{\lambda_2}\left(\|\boldsymbol{\theta}\|_{\boldsymbol{W}_l}\right) + \eta \sum_{k=0}^{q} \boldsymbol{\theta}_k^\top \boldsymbol{V} \boldsymbol{\theta}_k. \end{aligned} \tag{6}$$

When incorporating the discrete realizations $\widetilde{\boldsymbol{X}}_i$'s into the objective function (6), SNPinter performs partially functional interaction regression analysis with local sparsity by minimizing the penalized objective function:

$$\begin{aligned} Q(\boldsymbol{\theta}, \alpha, \boldsymbol{\gamma}) =& \frac{1}{2n} \sum_{i=1}^{n} \Bigg\{ y_i - \alpha - \sum_{k=1}^{q} z_{ik}\gamma_k - \left[\widetilde{\boldsymbol{X}}_i^\top \boldsymbol{\Omega}_i \left(\boldsymbol{\Omega}_i^\top \boldsymbol{\Omega}_i\right)^{-1} \int_0^T \boldsymbol{\phi}(t)\boldsymbol{\psi}(t)^\top \mathrm{d}t \right] \boldsymbol{\theta}_0 \\ & \qquad - \sum_{k=1}^{q} z_{ik} \left[\left(\widetilde{\boldsymbol{X}}_i^\top \boldsymbol{\Omega}_i \left(\boldsymbol{\Omega}_i^\top \boldsymbol{\Omega}_i\right)^{-1} \int_0^T \boldsymbol{\phi}(t)\boldsymbol{\psi}(t)^\top \mathrm{d}t \right) \boldsymbol{\theta}_k \right] \Bigg\}^2 \\ & \qquad + \sum_{k=1}^{q} \sum_{l=1}^{M_n} p_{\lambda_1}\left(\|\boldsymbol{\theta}_k\|_{\boldsymbol{W}_l}\right) + \sum_{l=1}^{M_n} p_{\lambda_2}\left(\|\boldsymbol{\theta}\|_{\boldsymbol{W}_l}\right) + \eta \sum_{k=0}^{q} \boldsymbol{\theta}_k^\top \boldsymbol{V} \boldsymbol{\theta}_k. \end{aligned}$$

Let $\boldsymbol{U} = (\boldsymbol{u}_1, \ldots, \boldsymbol{u}_n)^\top$ be an $n \times L_\beta$ matrix, where the (i,j)th entry is $u_{ij} = \widetilde{\boldsymbol{X}}_i^\top \boldsymbol{\Omega}_i \left(\boldsymbol{\Omega}_i^\top \boldsymbol{\Omega}_i\right)^{-1} \int_0^T \boldsymbol{\phi}(t)\psi_j(t)\mathrm{d}t$. Here, $\boldsymbol{u}_i = \left(u_{i1}, \ldots, u_{iL_\beta}\right)^\top$ is an L_β-vector. Additionally, define $\widetilde{\boldsymbol{U}} = (\widetilde{\boldsymbol{u}}_1, \ldots, \widetilde{\boldsymbol{u}}_n)^\top$ as the n by $q \times L_\beta$ matrix, with $\widetilde{\boldsymbol{u}}_i = \boldsymbol{Z}_i \otimes \boldsymbol{u}_i$. Let $\boldsymbol{R} = \left(\boldsymbol{U}, \widetilde{\boldsymbol{U}}\right)$, which is an $n \times q_n$ matrix and $q_n = (q+1) \times L_\beta$. With these definitions, the objective function can be more concisely and intuitively expressed as:

$$Q(\boldsymbol{\theta}, \alpha, \boldsymbol{\gamma}) = \frac{1}{2n}\sum_{i=1}^{n}\left(y_i - \alpha - \boldsymbol{Z}_i^\top\boldsymbol{\gamma} - \boldsymbol{R}_i^\top\boldsymbol{\theta}\right)^2 + \sum_{k=1}^{q}\sum_{l=1}^{M_n} p_{\lambda_1}\left(\|\boldsymbol{\theta}_k\|_{\boldsymbol{W}_l}\right) + \sum_{l=1}^{M_n} p_{\lambda_2}\left(\|\boldsymbol{\theta}\|_{\boldsymbol{W}_l}\right) + \eta\sum_{k=0}^{q}\boldsymbol{\theta}_k^\top \boldsymbol{V}\boldsymbol{\theta}_k. \tag{7}$$

1.3.2 Computation

We develop an iterative algorithm based on the local quadratic approximation (LQA) technique, which optimizes objective function (8) by approximating through the calculation of its first and second derivatives at a given point:

$$\min_{\boldsymbol{\theta},\alpha,\boldsymbol{\gamma}}\left\{\frac{1}{2n}\sum_{i=1}^{n}\left(y_i - \alpha - \boldsymbol{Z}_i^\top\boldsymbol{\gamma} - \boldsymbol{R}_i^\top\boldsymbol{\theta}\right)^2 + \sum_{k=1}^{q}\sum_{l=1}^{M_n} p_{\lambda_1}\left(\|\boldsymbol{\theta}_k\|_{\boldsymbol{W}_l}\right) + \sum_{l=1}^{M_n} p_{\lambda_2}\left(\|\boldsymbol{\theta}\|_{\boldsymbol{W}_l}\right) + \eta\sum_{k=0}^{q}\boldsymbol{\theta}_k^\top \boldsymbol{V}\boldsymbol{\theta}_k\right\}. \tag{8}$$

At the $(s+1)$th iteration, with estimate $\boldsymbol{\theta}_k^{(s)}$ from the sth iteration, we have the local quadratic approximation for the penalty term $p_{\lambda_1}(\cdot)$:

$$\begin{aligned} p_{\lambda_1}\left(\|\boldsymbol{\theta}_k\|_{\boldsymbol{W}_l}\right) &\approx p_{\lambda_1}(\|\boldsymbol{\theta}_k^{(s)}\|_{\boldsymbol{W}_l}) + \frac{1}{2}\frac{p'_{\lambda_1}(\|\boldsymbol{\theta}_k^{(s)}\|_{\boldsymbol{W}_l})}{\|\boldsymbol{\theta}_k^{(s)}\|_{\boldsymbol{W}_l}}(\|\boldsymbol{\theta}_k,\|_{\boldsymbol{W}_l}^2 - \|\boldsymbol{\theta}_k^{(s)}\|_{\boldsymbol{W}_l}^2), \\ &= \frac{1}{2}\frac{p'_{\lambda_1}(\|\boldsymbol{\theta}_k^{(s)}\|_{\boldsymbol{W}_l})}{\|\boldsymbol{\theta}_k^{(s)}\|_{\boldsymbol{W}_l}}\|\boldsymbol{\theta}_k\|_{\boldsymbol{W}_l}^2 + G_0(\boldsymbol{\theta}_k^{(s)}), \end{aligned}$$

where $p'_{\lambda_1}(t) = \lambda_1\left(1 - |t|/\left(\lambda_1\xi\right)\right)_+$ is the first order derivative of $p_{\lambda_1}(t)$, and $G_0(\boldsymbol{\theta}_k^{(s)})$ is a function of $\boldsymbol{\theta}_k^{(s)}$ that does not depend on $\boldsymbol{\theta}_k$. Similarly, for the penalty term $p_{\lambda_2}(\cdot)$, we have:

$$\begin{aligned} p_{\lambda_2}\left(\|\boldsymbol{\theta}\|_{\boldsymbol{W}_l}\right) &\approx p_{\lambda_2}(\|\boldsymbol{\theta}^{(s)}\|_{\boldsymbol{W}_l}) + \frac{1}{2}\frac{p'_{\lambda_1}(\|\boldsymbol{\theta}^{(s)}\|_{\boldsymbol{W}_l})}{\|\boldsymbol{\theta}^{(s)}\|_{\boldsymbol{W}_l}}(\|\boldsymbol{\theta}\|_{\boldsymbol{W}_l}^2 - \|\boldsymbol{\theta}^{(s)}\|_{\boldsymbol{W}_l}^2), \\ &= \frac{1}{2}\frac{p'_{\lambda_1}(\|\boldsymbol{\theta}^{(s)}\|_{\boldsymbol{W}_l})}{\|\boldsymbol{\theta}^{(s)}\|_{\boldsymbol{W}_l}}\|\boldsymbol{\theta}\|_{\boldsymbol{W}_l}^2 + G_1(\boldsymbol{\theta}^{(s)}), \end{aligned}$$

where $G_1(\boldsymbol{\theta}^{(s)})$ is a function of $\boldsymbol{\theta}^{(s)}$ that does not depend on $\boldsymbol{\theta}$. As such, we can obtain the LQA approximation of the sparse group penalty as:

$$\sum_{k=1}^{q}\sum_{l=1}^{M_n} p_{\lambda_1}\left(\|\boldsymbol{\theta}_k\|_{\boldsymbol{W}_l}\right) + \sum_{l=1}^{M_n} p_{\lambda_2}\left(\|\boldsymbol{\theta}\|_{\boldsymbol{W}_l}\right) \approx \boldsymbol{\theta}^\top \breve{\boldsymbol{W}}^{(s)}\boldsymbol{\theta} + G(\boldsymbol{\theta}^{(s)}),$$

where $\breve{\boldsymbol{W}}^{(s)} = \text{diag}\left(\breve{\boldsymbol{W}}_0^{(s)}, \ldots, \breve{\boldsymbol{W}}_q^{(s)}\right)$ is a $q_n \times q_n$ block diagonal matrix with

$$\breve{\boldsymbol{W}}_0^{(s)} = \frac{1}{2}\sum_{l=1}^{M_n} \frac{p'_{\lambda_2}(\|\boldsymbol{\theta}^{(s)}\|_{\boldsymbol{W}_l})}{\|\boldsymbol{\theta}^{(s)}\|_{\boldsymbol{W}_l}} \boldsymbol{W}_l,$$

and

$$\breve{\boldsymbol{W}}_k^{(s)} = \frac{1}{2}\sum_{l=1}^{M_n} \left(\frac{p'_{\lambda_1}(\|\boldsymbol{\theta}_k^{(s)}\|_{\boldsymbol{W}_l})}{\|\boldsymbol{\theta}_k^{(s)}\|_{\boldsymbol{W}_l}} + \frac{p'_{\lambda_2}(\|\boldsymbol{\theta}^{(s)}\|_{\boldsymbol{W}_l})}{\|\boldsymbol{\theta}^{(s)}\|_{\boldsymbol{W}_l}} \right) \boldsymbol{W}_l,$$

for each $k = 1, \ldots, q$. In addition, $G(\boldsymbol{\theta}^{(s)})$ consists of $G_0(\boldsymbol{\theta}_k^{(s)})$'s and $G_1(\boldsymbol{\theta}^{(s)})$'s, is independent of $\boldsymbol{\theta}$.

Let $\boldsymbol{D}$ be an $n \times p_n$ matrix with the ith row $\boldsymbol{D}_i$ being $(1, \boldsymbol{Z}_i^\top, \boldsymbol{u}_i^\top, \breve{\boldsymbol{u}}_i^\top)^\top \in \mathbb{R}^{p_n}$ and $p_n = q_n + q + 1$. Denote $\boldsymbol{\omega} = (\alpha, \boldsymbol{\gamma}^\top, \boldsymbol{\theta}^\top)^\top \in \mathbb{R}^{p_n}$ as the vector of unknown parameters. Moreover, let $\widetilde{\boldsymbol{V}} = \text{diag}(\boldsymbol{0}_{q+1}, \boldsymbol{V}, \ldots, \boldsymbol{V})$ and $\widetilde{\boldsymbol{W}}^{(s)} = \text{diag}(\boldsymbol{0}_{q+1}, \breve{\boldsymbol{W}}^{(s)})$ be $p_n \times p_n$ matrices, where $\boldsymbol{0}_{q+1}$ is a $(q+1) \times (q+1)$ matrix with all elements being 0. The overall objective function at the $(s+1)$th iteration is:

$$\widetilde{Q}(\boldsymbol{\omega}|\boldsymbol{\omega}^{(s)}) = \frac{1}{2n}\sum_{i=1}^{n} \left(y_i - \boldsymbol{D}_i^\top \boldsymbol{\omega}\right)^2 + \boldsymbol{\omega}^\top \widetilde{\boldsymbol{W}}^{(s)} \boldsymbol{\omega} + \eta \boldsymbol{\omega}^\top \widetilde{\boldsymbol{V}} \boldsymbol{\omega}. \tag{9}$$

Set the first order derivative of $\widetilde{Q}(\boldsymbol{\omega}|\boldsymbol{\omega}^{(s)})$ with respect to $\boldsymbol{\omega}$ equal to 0:

$$\frac{\partial \widetilde{Q}(\boldsymbol{\omega}|\boldsymbol{\omega}^{(s)})}{\partial \boldsymbol{\omega}} = \frac{1}{n}\boldsymbol{D}^\top(\boldsymbol{D}\boldsymbol{\omega} - \boldsymbol{y}) + 2\widetilde{\boldsymbol{W}}^{(s)}\boldsymbol{\omega} + 2\eta\widetilde{\boldsymbol{V}}\boldsymbol{\omega} = 0,$$

where $\boldsymbol{y} = (y_1, \ldots, y_n)^\top$. Then, we have:

$$\boldsymbol{\omega}^{(s+1)} = (\boldsymbol{D}^\top\boldsymbol{D} + 2n\widetilde{\boldsymbol{W}}^{(s)} + 2n\eta\widetilde{\boldsymbol{V}})^{-1}\boldsymbol{D}^\top\boldsymbol{y}. \tag{10}$$

Overall, the proposed computational algorithm is summarized as follows:

Algorithm 1 Computational algorithm for SNPinter in (8).

1: Initialize $\boldsymbol{\omega}$ as $\boldsymbol{\omega}^{(0)} = (\boldsymbol{D}^\top\boldsymbol{D} + 2n\eta\widetilde{\boldsymbol{V}})^{-1}\boldsymbol{D}^\top\boldsymbol{y}$.

2: Repeat: for $s = 0, 1, 2, \ldots$, update:

$$\boldsymbol{\omega}^{(s+1)} = (\boldsymbol{D}^\top\boldsymbol{D} + 2n\widetilde{\boldsymbol{W}}^{(s)} + 2n\eta\widetilde{\boldsymbol{V}})^{-1}\boldsymbol{D}^\top\boldsymbol{y},$$

until convergence, where we use the norm of the difference between two consecutive estimates smaller than a pre-specified threshold as the criterion for convergence.

3: The final estimates $\widehat{\alpha}$, $\widehat{\boldsymbol{\gamma}}$, $\widehat{\beta}_k(t) = \boldsymbol{\psi}(t)^\top\widehat{\boldsymbol{\theta}}_k$ can then be obtained from $\widehat{\boldsymbol{\omega}}$.

1.3.3 Tuning parameter selection

As argued in the literature, the value of M_n is not crucial, as the smoothness of estimation is primarily controlled by the roughness penalty, as opposed to the number of knots. In our numerical study, we set M_n as 71. Following the published studies (Shi et al., 2014; Wu et al., 2020), we set $\lambda_2 = \sqrt{q+1}\lambda_1$ and $\xi = 6$ and subsequently conduct a grid search to determine the optimal values of (η, λ_1) based on prediction performance.

2 Software development

The FunctanSNP package contains two main functions, SNPlm and SNPinter, designed for performing partially functional linear and interaction analysis, respectively. Additionally, to facilitate a better understanding of the models/approaches and future numerical studies, we have also developed a function for generating simulated data, with which users can specify the number of genetic variants m and the number of samples n. The simulated data generation process is described in Section 3. With simple modifications, users will be able to generate data under other models. The simulation function `simX` is illustrated as follows.

```
> library(FunctanSNP)
> ### Data visualization
> simdata <- simX(n = 20, m = 50, seed = 1, d.ratio = 0)
> location <- simdata$location
> X <- simdata$X[1:2, ]
> SNPgvfres <- SNPgvf(location, X, type = "Bspline", nbasis = 15, params = 4,
+                     Plot = FALSE)
> par(mfrow = c(1,2))
> plotRawdata(location, X, color = 1:2)
> plotGVF(SNPgvfres)
```

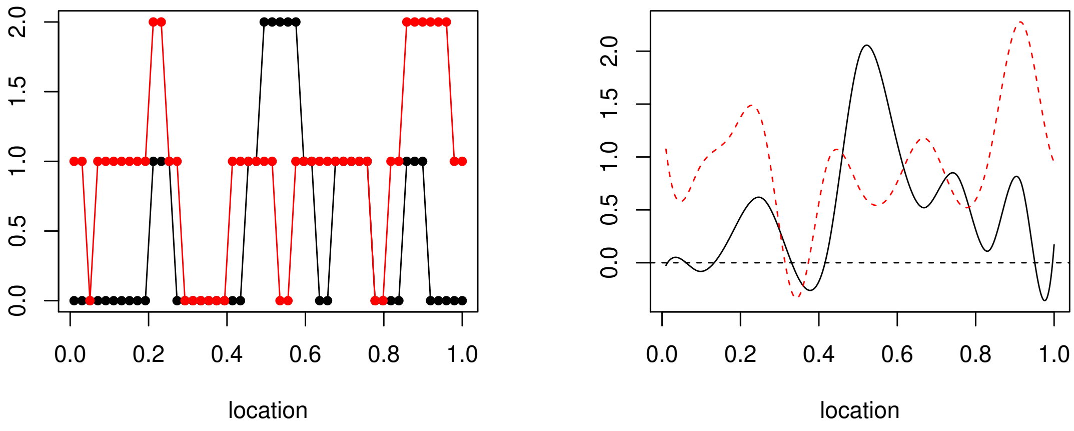

Figure 1: Simulated data for genotypes of two subjects. Left: discrete realization. Right: genetic variant function.

2.1 SNPlm

When using the `SNPlm` function, the quantitative trait `y` needs to be organized as a vector, the covariates `z` need to be organized as a matrix, and the genetic variants need to be specified by a position vector `location` and a corresponding genotype matrix `X`. For the genetic variant function $X_i(t)$ and the genetic effect function $\beta(t)$, users can choose different types (`type`) and numbers (`nbasis`) of basis functions, where `1` corresponds to the relevant parameters for $X_i(t)$ and `2` corresponds to $\beta(t)$. Below, we provide an example to demonstrate how to use these options.

```
> ### Partially functional linear regression analysis
> simdata1 <- simData1(n = 300, m = 100, seed = 1)
> y <- simdata1$y[1:200]
> z <- simdata1$z[1:200, ]
> X <- simdata1$X[1:200, ]
> location <- simdata1$location
> SNPlmres <- SNPlm(y, z, location, X, type1 = "Bspline", type2 = "Bspline",
+                     nbasis1 = 5, nbasis2 = 5, params1 = 4, params2 = 4,
+                     intercept = FALSE, Plot = FALSE)
```

```
>
> ### Extract coefficients from an "SNPlm" object
> coefSNPlm <- coefSNPreg(x = SNPlmres)
> coefSNPlm$alpha #Intercept
[1] 0
> coefSNPlm$gamma #Coefficients of scalar variables
       Z1        Z2
0.4109868 0.7942781
> coefSNPlm$b     #Coefficients of basis functions
        H1          H2          H3          H4          H5
-4.3185268 -0.5489726  0.5008260  0.2794757  3.9342351
> class(coefSNPlm$betat)
[1] "fd"
>
> ### Make predictions from an "SNPlm" object
> newz <- simdata1$z[-(1:200), ]
> newX <- simdata1$X[-(1:200), ]
> predicted.SNPlm <- predictSNPreg(x = SNPlmres, newz = newz, newX = newX)
>
> ### Visualization
> par(mfrow = c(1,2))
> plotSNPreg(x = SNPlmres, type = "beta")
> plotSNPreg(x = SNPlmres, type = "residuals")
```

The graphical outputs consisting of the genetic effect function $\beta(t)$ and fitted residuals are as follows.

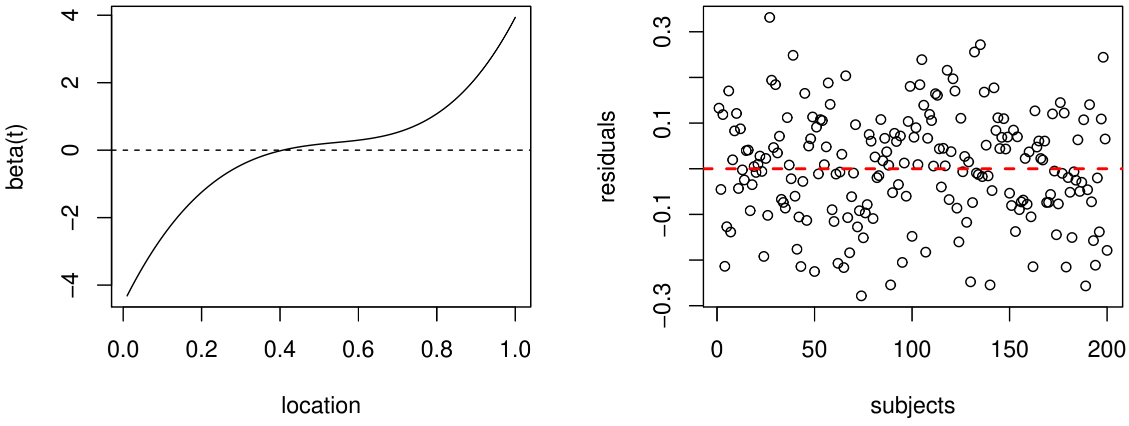

Figure 2: Sample output of SNPlm. Left: $\beta(t)$. Right: residuals.

2.2 Partially functional interaction analysis

The usage of functions `SNPlm` and `SNPinter` is similar, making them easy for users. One key difference is that `SNPlm` requires users to specify the tuning parameters λ_1, λ_2, and η in advance. To facilitate this, users can specify the ranges of these tuning parameters using the variables `lambda1`, `lambda2`, and `eta`, and subsequently determine the optimal parameters using the cross-validation function `SNPcvinter`. These functions are demonstrated below.

```
> ### Partially functional interaction analysis
> simdata2 <- simData2(n = 300, m = 100, seed = 1)
> y <- simdata2$y[1:200]
> z <- simdata2$z[1:200, ]
> X <- simdata2$X[1:200, ]
> location <- simdata2$location
> lambda1 <- c(0.05, 0.1)
> lambda2 <- sqrt(3)*lambda1
> eta <- c(1e-10, 1e-12)
> ### Cross validation
> SNPcvinterres <- SNPcvinter(y, z, location, X, K = 3, lambda1, lambda2, eta,
+                                 type1 = "Bspline", nbasis1 = 5, params1 = 4,
```

```
+                              Bsplines = 10, norder = 4, intercept = FALSE,
+                              eps = 1e-2, maxstep = 1e2, Plot = FALSE)
> cat("The selected lambda1 is: ", SNPcvinterres$lambda1Select)
The selected lambda1 is:  0.05
> cat("The selected lambda2 is: ", SNPcvinterres$lambda2Select)
The selected lambda2 is:  0.08660254
> cat("The selected eta is: ", SNPcvinterres$etaSelect)
The selected eta is:  1e-12
> SNPinterres <- SNPinter(y, z, location, X, lambda1 = SNPcvinterres$lambda1Select,
+                         lambda2 = SNPcvinterres$lambda2Select,
+                         eta = SNPcvinterres$etaSelect, type1 = "Bspline",
+                         nbasis1 = 5, params1 = 4, Bsplines = 10, norder = 4,
+                         intercept = FALSE, eps = 1e-2, maxstep = 1e2, Plot = FALSE)
>
> ### Extract coefficients from a "SNPinter" object
> coefSNPinter <- coefSNPreg(x = SNPinterres)
> coefSNPinter$alpha #Intercept
[1] 0
> coefSNPinter$gamma #Coefficients of scalar variables
[1] 0.4023001 0.8046291
> coefSNPinter$b     #Coefficients of basis functions
$b0
 [1]  2.283537e+00 -8.936319e+00  2.426975e-04  0.000000e+00 -3.000076e-05
 [6]  1.175947e-04 -5.689166e-04  5.987086e-03  8.534932e+00 -1.906314e+00


$b1
 [1]  1.933577 -8.243376  0.000000  0.000000  0.000000  0.000000
 [7]  0.000000  0.000000  0.000000  0.000000


$b2
 [1] -9.770284e-05  0.000000e+00  0.000000e+00  0.000000e+00  0.000000e+00
```

```
 [6]  0.000000e+00  0.000000e+00  0.000000e+00  5.855385e+00  2.305561e+00
>
> ### Make prediction from a "SNPlm" object
> newz <- simdata2$z[-(1:200), ]
> newX <- simdata2$X[-(1:200), ]
> predicted.SNPinter <- predictSNPreg(x = SNPinterres, newz = newz, newX = newX)
>
> ### Visualization
> par(mfrow = c(2,2))
> plotSNPreg(x = SNPinterres, type = "beta")
> plotSNPreg(x = SNPinterres, type = "residuals")
```

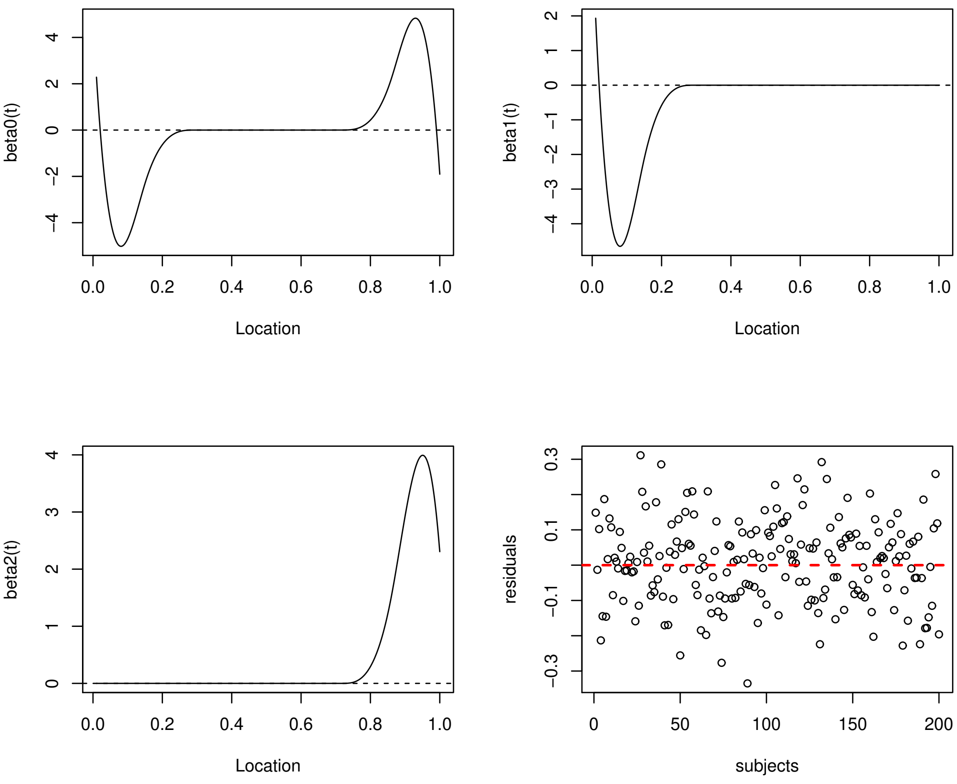

Figure 3: Sample output of SNPinter.

3 Simulation

We conduct simulations to gain a deeper understanding of the analysis and "validate" the functions in the package.

3.1 SNPlm

We generate data from the following model:

$$y_i = \sum_{k=1}^{2} z_{ik}\gamma_k + \widetilde{\boldsymbol{X}}_i^\top \boldsymbol{\Omega}_i \left(\boldsymbol{\Omega}_i^\top \boldsymbol{\Omega}_i\right)^{-1} \int_0^T \boldsymbol{\phi}(t)\beta(t)\mathrm{d}t + \varepsilon_i,$$

where the scalar covariates z_k for $k = 1, 2$ are independently generated from the standard normal distribution, with the corresponding coefficient vector $\boldsymbol{\gamma} = (0.4, 0.8)^\top$. Additionally, ε_i follows a normal distribution $N(0, 0.1)$. We then generate sequence genotypes $\widetilde{\boldsymbol{X}}_i = (X_i(t_{i1}), \ldots, X_i(t_{im_i}))^\top$ by solving the optimization problem:

$$\min_{\widetilde{\boldsymbol{X}}_i} \sum_{j=1}^{m_i} \left\{ \widetilde{\boldsymbol{X}}_i(t_{ij}) - X_i^*(t_{ij}) \right\}^2,$$
$$\text{s.t. } X_i(t_{ij}) \in \{0, 1, 2\} \text{ for each } j = 1, \ldots, m.$$

Here, $X_i^*(t)$ is generated as $X_i^*(t) = \sum_j a_{ij}\phi_j(t)$, where a_{ij} follows a normal distribution with mean 1 and standard deviation 1. Each $\phi_j(t)$ is a B-spline basis function with order 4 and 71 equally spaced knots. For the genetic effect function $\beta(t) = \beta_0(t)$, we consider

$$\beta_0(t) = \begin{cases} -36(t-0.3)^2, & 0 \le t < 0.3, \\ 0, & 0.3 \le t < 0.7, \\ 36(t-0.7)^2, & 0.7 \le t \le 1. \end{cases}$$

and generate the response

$$y_i = \alpha + \sum_{k=1}^{2} z_{ik}\gamma_k + \sum_{j=1}^{m_i} X_i(t_{ij})\beta(t_{ij}) + \varepsilon_i.$$

In all simulations, we consider two cases:

(a) Case I, where $m_i = m$ and $0 < t_1 < t_2 < \ldots < t_m = 1$ with $m = 100$ equally spaced points on $(0, 1]$.

(b) Case II, with 35% random missing values for each $X_i(t)$ based on Case I.

For comparison, we consider two alternatives: (a) Alt.1 adopts a parametric (as opposed to functional) model; and (b) Alt.2 takes individual genetic variants as covariates and assumes that the effects are functions. Here, it is noted that advantages of the functional modeling-based approaches have been established elsewhere (based on, for example, extensive numerical comparisons), and that the goal of simulation and comparison here is for the completeness of this study. We evaluate performance using the following criteria: (a) Integrated squared errors (ISE_β): $\mathrm{ISE}_\beta = \frac{1}{l_\mathcal{T}} \int_\mathcal{T} (\widehat{\beta}(t) - \beta(t))^2 dt$, where $l_\mathcal{T}$ is the length of the region. (b) Root mean squared errors of $\boldsymbol{\gamma}$ ($\mathrm{RMSE}_{\boldsymbol{\gamma}}$): $\mathrm{RMSE}_{\boldsymbol{\gamma}} = \|\widehat{\boldsymbol{\gamma}} - \boldsymbol{\gamma}\|_2$. It is observed that SNPlm performs significantly better than the two alternative methods in estimating the genetic effect function $\beta(t)$, and the estimation for the scalar effect $\boldsymbol{\gamma}$ is more stable and consistent.

Table 1: Simulation results of SNPlm: mean (sd) based on 500 replicates.

		Case I			Case II		
		Alt.1	Alt.2	SNPlm	Alt.1	Alt.2	SNPlm
$n = 200$	ISE_β	1.1141	1.1125	0.1697	1.1141	1.1226	0.3578
		(0.001)	(0.001)	(0.051)	(0.001)	(0.000)	(0.136)
	$\mathrm{RMSE}_\gamma \times 10^2$	1.2160	0.9012	0.9012	1.216	1.4684	1.0830
		(0.636)	(0.445)	(0.445)	(0.636)	(0.715)	(0.565)
$n = 500$	ISE_β	1.1126	1.1124	0.1428	1.1126	1.1226	0.3563
		(0.000)	(0.000)	(0.033)	(0.000)	(0.000)	(0.115)
	$\mathrm{RMSE}_\gamma \times 10^2$	0.6150	0.5631	0.5631	0.6150	0.9076	0.6893
		(0.340)	(0.299)	(0.299)	(0.340)	(0.480)	(0.339)

3.2 SNPinter

We generate data using the following model:

$$y_i = \sum_{k=1}^{2} z_{ik}\gamma_k + \widetilde{\boldsymbol{X}}_i^\top \boldsymbol{\Omega}_i \left(\boldsymbol{\Omega}_i^\top \boldsymbol{\Omega}_i\right)^{-1} \int_0^T \boldsymbol{\phi}(t)\beta_0(t)\mathrm{d}t + \sum_{k=1}^{2} z_{ik} \left\{ \widetilde{\boldsymbol{X}}_i^\top \boldsymbol{\Omega}_i \left(\boldsymbol{\Omega}_i^\top \boldsymbol{\Omega}_i\right)^{-1} \int_0^T \boldsymbol{\phi}(t)\beta_k(t)dt \right\} + \varepsilon_i,$$

where z_{ik}, $\boldsymbol{\gamma}$, ε_i, $\widetilde{\boldsymbol{X}}_i$, X_i^*, and $\beta_0(t)$ are generated in the same way as above. For the functional interaction effects $\beta_1(t)$ and $\beta_2(t)$, we consider: (a) $\beta_1(t) = \beta_0(t)$ for $t \in (0, 0.3]$, and $\beta_1(t) = 0$ otherwise, (b) $\beta_2(t) = \beta_0(t)$ for $t \in (0.7, 1]$, and $\beta_2(t) = 0$ otherwise. These functions satisfy the "main effect, interaction" hierarchy and are plotted in Figure 4.

For comparison, we consider the following alternatives: (a) Alt.1 adopts a parametric interaction model and conducts least squares estimation; (b) Alt.2 adopts a parametric interaction model and applies MCP for selection, which may not respect the selection hierarchy; (c) Alt.3 adopts a parametric interaction model and applies MCP+group MCP for selection, which respects the selection hierarchy; (d) Alt.4 takes individual genetic variants and corresponding interactions as covariates. The genetic main and interaction effects are assumed to be functions. A smoothness penalty, which has the same form as the third penalty term in (5), is applied; (e) Alt.5 adopts the same model as Alt.4. It applies a smoothness penalty and a functional MCP penalty, which have the same form as the third and first penalty terms in (5); (f) Alt.6 adopts the same model as Alt.4 and the same penalty as the proposed approach; (g) Alt.7 adopts the same model as the proposed approach and only the smoothness penalty; and (h) Alt.8 adopts the same model as the proposed approach and only the smoothness and functional MCP penalties.

Performance is evaluated using the following criteria: (a) Integrated squared errors on null region (ISE_0): $\mathrm{ISE}_{0k} = \frac{1}{l_{\mathcal{I}_{0k}}} \int_{\mathcal{I}_0} (\widehat{\beta}_k(t) - \beta_k(t))^2 dt$, where $l_{\mathcal{I}_{0k}}$is the length of null region $\mathcal{I}_k$ of $\beta_k(t)$, (b) Integrated squared errors on nonnull region (ISE_1): $\mathrm{ISE}_{1k} = \frac{1}{l_{\mathcal{I}^c_{1k}}} \int_{\mathcal{I}^c_1} (\widehat{\beta}_k(t) - \beta_k(t))^2 dt$, where $l_{\mathcal{I}^c_{1k}}$is the length of nonnull region $\mathcal{I}^c_k$ of $\beta_k(t)$, (c) Root mean squared errors of $\boldsymbol{\gamma}$ ($\mathrm{RMSE}_{\boldsymbol{\gamma}}$): $\mathrm{RMSE}_{\boldsymbol{\gamma}} = \|\widehat{\boldsymbol{\gamma}} - \boldsymbol{\gamma}\|_2$, (d) Average proportion of null regions that are correctly identified (PNRCI), which is the functional counterpart of true negative rate in parametric variable selection, and (e) Average proportion of nonnull regions that are falsely identified (PNRFI), which is the functional counterpart of false negative rate in parametric variable selection.

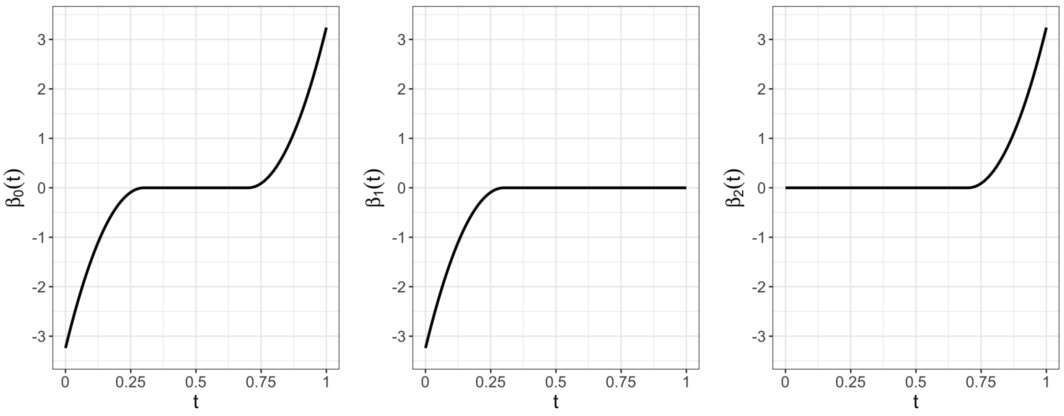

Figure 4: Simulation: (a) $\beta_0(t)$, (b) $\beta_1(t)$, (c) $\beta_2(t)$.

Table 2: Comparison of estimation performance under different sample sizes: mean (sd) based on 500 replicates.

		Alt.1	Alt.2	Alt.3	Alt.4	Alt.5	Alt.6	Alt.7	Alt.8	SNPinter
						Case I & $n = 200$				
$\mathrm{ISE}_0 \times 10$	β_0	0.3696	0.4901	0.0417	0.0069	0.0080	0.0054	1.2678	1.2441	0.2258
		(0.000)	(0.000)	(0.000)	(0.000)	(0.000)	(0.003)	(0.000)	(0.000)	(0.185)
	β_1	0.4685	0.0195	0.0260	0.0116	0.0074	0.0071	1.3433	0.4805	0.3200
		(0.080)	(0.057)	(0.026)	(0.005)	(0.006)	(0.005)	(0.452)	(0.643)	(0.523)
	β_2	0.4654	0.0212	0.0233	0.0111	0.0068	0.0068	1.2092	0.5101	0.3296
		(0.078)	(0.052)	(0.026)	(0.005)	(0.005)	(0.006)	(0.426)	(0.664)	(0.578)
ISE_1	β_0	1.1132	1.1141	1.1142	1.1131	1.1129	1.1135	0.1841	0.1916	0.3574
		(0.001)	(0.001)	(0.002)	(0.001)	(0.001)	(0.001)	(0.051)	(0.057)	(0.203)
	β_1	0.7528	0.7539	0.7547	0.7533	0.7537	0.7539	0.1263	0.2591	0.2907
		(0.002)	(0.002)	(0.002)	(0.001)	(0.001)	(0.001)	(0.053)	(0.137)	(0.165)
	β_2	0.8207	0.8203	0.8209	0.8194	0.8197	0.8200	0.1167	0.2621	0.2991
		(0.002)	(0.002)	(0.002)	(0.001)	(0.001)	(0.001)	(0.052)	(0.131)	(0.154)
$\mathrm{RMSE} \times 10$	γ	0.3914	0.2993	0.4122	0.1984	0.2144	0.2447	0.1949	0.3056	0.3678
		(0.214)	(0.186)	(0.218)	(0.100)	(0.127)	(0.141)	(0.100)	(0.205)	(0.250)
						Case I & $n = 500$				
$\mathrm{ISE}_0 \times 10$	β_0	0.2724	0.2213	0.0851	0.0053	0.0061	0.0034	1.3221	1.2947	0.1696
		(0.000)	(0.000)	(0.000)	(0.000)	(0.000)	(0.003)	(0.000)	(0.000)	(0.190)
	β_1	0.4008	0.0695	0.0733	0.0083	0.0050	0.0045	1.0812	0.2727	0.1844
		(0.055)	(0.094)	(0.092)	(0.004)	(0.004)	(0.004)	(0.265)	(0.401)	(0.343)
	β_2	0.4033	0.0711	0.0729	0.0081	0.0046	0.0042	0.9195	0.2882	0.1724
		(0.057)	(0.096)	(0.092)	(0.004)	(0.004)	(0.004)	(0.249)	(0.490)	(0.336)
ISE_1	β_0	1.1127	1.1127	1.1127	1.1127	1.1126	1.1130	0.1535	0.1569	0.2887
		(0.001)	(0.001)	(0.001)	(0.000)	(0.001)	(0.001)	(0.024)	(0.028)	(0.157)
	β_1	0.7529	0.7527	0.7529	0.7530	0.7532	0.7535	0.0993	0.2063	0.2210
		(0.001)	(0.001)	(0.001)	(0.000)	(0.001)	(0.001)	(0.031)	(0.103)	(0.114)
	β_2	0.8197	0.8195	0.8196	0.8192	0.8194	0.8197	0.0828	0.2216	0.2437
		(0.001)	(0.001)	(0.001)	(0.000)	(0.001)	(0.001)	(0.027)	(0.107)	(0.119)
$\mathrm{RMSE} \times 10$	γ	0.1755	0.1460	0.1763	0.1203	0.1316	0.1612	0.1200	0.2213	0.2480
		(0.092)	(0.079)	(0.102)	(0.062)	(0.073)	(0.095)	(0.061)	(0.160)	(0.182)

Table 3: Comparison of selection performance under different sample sizes: mean (sd) based on 500 replicates.

		Alt.1	Alt.2	Alt.3	Alt.4	Alt.5	Alt.6	Alt.7	Alt.8	SNPinter
						Case I & $n = 200$				
PNRCI	β_0	0.0000	0.0000	0.0000	0.0000	0.0000	0.0351	0.0000	0.0000	0.7187
		(0.000)	(0.000)	(0.000)	(0.000)	(0.000)	(0.140)	(0.000)	(0.000)	(0.360)
	β_1	0.0000	0.0000	0.0000	0.0000	0.1958	0.1781	0.0000	0.7641	0.8529
		(0.000)	(0.000)	(0.000)	(0.000)	(0.272)	(0.261)	(0.000)	(0.271)	(0.225)
	β_2	0.0000	0.0000	0.0000	0.0000	0.2046	0.1832	0.0000	0.7539	0.8440
		(0.000)	(0.000)	(0.000)	(0.000)	(0.288)	(0.273)	(0.000)	(0.283)	(0.242)
PNRFI	β_0	0.0000	0.0000	0.0001	0.0000	0.0000	0.0030	0.0000	0.0000	0.1852
		(0.000)	(0.000)	(0.001)	(0.000)	(0.000)	(0.017)	(0.000)	(0.000)	(0.183)
	β_1	0.0000	0.0000	0.0001	0.0000	0.0264	0.0257	0.0000	0.2409	0.2733
		(0.000)	(0.000)	(0.001)	(0.000)	(0.083)	(0.074)	(0.000)	(0.165)	(0.195)
	β_2	0.0000	0.0000	0.0000	0.0000	0.0101	0.0112	0.0000	0.2270	0.2763
		(0.000)	(0.000)	(0.000)	(0.000)	(0.040)	(0.041)	(0.000)	(0.156)	(0.185)
						Case I & $n = 500$				
PNRCI	β_0	0.0000	0.0000	0.0000	0.0000	0.0000	0.0753	0.0000	0.0000	0.7499
		(0.000)	(0.000)	(0.000)	(0.000)	(0.000)	(0.210)	(0.000)	(0.000)	(0.349)
	β_1	0.0000	0.0000	0.0000	0.0000	0.2256	0.2257	0.0000	0.8253	0.8856
		(0.000)	(0.000)	(0.000)	(0.000)	(0.295)	(0.297)	(0.000)	(0.232)	(0.204)
	β_2	0.0000	0.0000	0.0000	0.0000	0.2434	0.2511	0.0000	0.7970	0.8885
		(0.000)	(0.000)	(0.000)	(0.000)	(0.319)	(0.317)	(0.000)	(0.260)	(0.202)
PNRFI	β_0	0.0000	0.0000	0.0000	0.0000	0.0000	0.0028	0.0000	0.0000	0.1898
		(0.000)	(0.000)	(0.000)	(0.000)	(0.000)	(0.014)	(0.000)	(0.000)	(0.165)
	β_1	0.0000	0.0000	0.0000	0.0000	0.0221	0.0225	0.0000	0.2413	0.2708
		(0.000)	(0.000)	(0.000)	(0.000)	(0.063)	(0.061)	(0.000)	(0.146)	(0.159)
	β_2	0.0000	0.0000	0.0000	0.0000	0.0108	0.0137	0.0000	0.2315	0.2690
		(0.000)	(0.000)	(0.000)	(0.000)	(0.036)	(0.040)	(0.000)	(0.152)	(0.164)

Table 4: Comparison of estimation performance under different cases: mean (sd) based on 500 replicates.

		Alt.1	Alt.2	Alt.3	Alt.4	Alt.5	Alt.6	Alt.7	Alt.8	SNPinter
						Case I & $n = 200$				
$\text{ISE}_0 \times 10$	β_0	0.3696	0.4901	0.0417	0.0069	0.0080	0.0054	1.2678	1.2441	0.2258
		(0.000)	(0.000)	(0.000)	(0.000)	(0.000)	(0.003)	(0.000)	(0.000)	(0.185)
	β_1	0.4685	0.0195	0.0260	0.0116	0.0074	0.0071	1.3433	0.4805	0.3200
		(0.080)	(0.057)	(0.026)	(0.005)	(0.006)	(0.005)	(0.452)	(0.643)	(0.523)
	β_2	0.4654	0.0212	0.0233	0.0111	0.0068	0.0068	1.2092	0.5101	0.3296
		(0.078)	(0.052)	(0.026)	(0.005)	(0.005)	(0.006)	(0.426)	(0.664)	(0.578)
ISE_1	β_0	1.1132	1.1141	1.1142	1.1131	1.1129	1.1135	0.1841	0.1916	0.3574
		(0.001)	(0.001)	(0.002)	(0.001)	(0.001)	(0.001)	(0.051)	(0.057)	(0.203)
	β_1	0.7528	0.7539	0.7547	0.7533	0.7537	0.7539	0.1263	0.2591	0.2907
		(0.002)	(0.002)	(0.002)	(0.001)	(0.001)	(0.001)	(0.053)	(0.137)	(0.165)
	β_2	0.8207	0.8203	0.8209	0.8194	0.8197	0.8200	0.1167	0.2621	0.2991
		(0.002)	(0.002)	(0.002)	(0.001)	(0.001)	(0.001)	(0.052)	(0.131)	(0.154)
$\text{RMSE} \times 10$	$\boldsymbol{\gamma}$	0.3914	0.2993	0.4122	0.1984	0.2144	0.2447	0.1949	0.3056	0.3678
		(0.214)	(0.186)	(0.218)	(0.100)	(0.127)	(0.141)	(0.100)	(0.205)	(0.250)
						Case II & $n = 200$				
$\text{ISE}_0 \times 10$	β_0	0.0713	0.0646	0.0073	0.0049	0.0049	0.0021	0.9576	0.9368	0.2187
		(0.000)	(0.000)	(0.000)	(0.000)	(0.000)	(0.199)	(0.000)	(0.000)	(0.212)
	β_1	0.1020	0.0211	0.0064	0.0098	0.0033	0.0029	1.3313	0.5071	0.3446
		(0.014)	(0.027)	(0.010)	(0.006)	(0.005)	(0.005)	(0.516)	(0.705)	(0.576)
	β_2	0.1011	0.0213	0.0067	0.0100	0.0031	0.0027	1.2531	0.4796	0.3427
		(0.015)	(0.030)	(0.012)	(0.006)	(0.005)	(0.005)	(0.512)	(0.696)	(0.592)
ISE_1	β_0	1.1223	1.1217	1.1232	1.1220	1.1220	1.1228	0.2409	0.2528	0.4578
		(0.001)	(0.001)	(0.000)	(0.001)	(0.001)	(0.001)	(0.084)	(0.092)	(0.245)
	β_1	0.7601	0.7605	0.7606	0.7598	0.7604	0.7604	0.1483	0.3291	0.3657
		(0.001)	(0.000)	(0.000)	(0.001)	(0.000)	(0.000)	(0.074)	(0.180)	(0.205)
	β_2	0.8266	0.8263	0.8266	0.8259	0.8264	0.8265	0.1845	0.3550	0.3847
		(0.001)	(0.001)	(0.000)	(0.001)	(0.001)	(0.001)	(0.087)	(0.165)	(0.185)
$\text{RMSE} \times 10$	$\boldsymbol{\gamma}$	1.1517	1.4636	1.1942	1.0651	1.1060	1.1064	0.2483	0.4672	0.5306
		(0.463)	(1.096)	(0.413)	(0.387)	(0.302)	(0.290)	(0.129)	(0.270)	(0.300)

Table 5: Comparison of selection performance under different cases: mean (sd) based on 500 replicates.

		Alt.1	Alt.2	Alt.3	Alt.4	Alt.5	Alt.6	Alt.7	Alt.8	SNPinter
						Case I & $n = 200$				
PNRCI	β_0	0.0000	0.0000	0.0000	0.0000	0.0000	0.0351	0.0000	0.0000	0.7187
		(0.000)	(0.000)	(0.000)	(0.000)	(0.000)	(0.140)	(0.000)	(0.000)	(0.360)
	β_1	0.0000	0.0000	0.0000	0.0000	0.1958	0.1781	0.0000	0.7641	0.8529
		(0.000)	(0.000)	(0.000)	(0.000)	(0.272)	(0.261)	(0.000)	(0.271)	(0.225)
	β_2	0.0000	0.0000	0.0000	0.0000	0.2046	0.1832	0.0000	0.7539	0.8440
		(0.000)	(0.000)	(0.000)	(0.000)	(0.288)	(0.273)	(0.000)	(0.283)	(0.242)
PNRFI	β_0	0.0000	0.0000	0.0001	0.0000	0.0000	0.0030	0.0000	0.0000	0.1852
		(0.000)	(0.000)	(0.001)	(0.000)	(0.000)	(0.017)	(0.000)	(0.000)	(0.183)
	β_1	0.0000	0.0000	0.0001	0.0000	0.0264	0.0257	0.0000	0.2409	0.2733
		(0.000)	(0.000)	(0.001)	(0.000)	(0.083)	(0.074)	(0.000)	(0.165)	(0.195)
	β_2	0.0000	0.0000	0.0000	0.0000	0.0101	0.0112	0.0000	0.2270	0.2763
		(0.000)	(0.000)	(0.000)	(0.000)	(0.040)	(0.041)	(0.000)	(0.156)	(0.185)
						Case II & $n = 200$				
PNRCI	β_0	0.0000	0.0000	0.0000	0.0000	0.0000	0.2547	0.0000	0.0000	0.6983
		(0.000)	(0.000)	(0.000)	(0.000)	(0.000)	(0.381)	(0.000)	(0.000)	(0.378)
	β_1	0.0000	0.0000	0.0000	0.0000	0.4525	0.4915	0.0000	0.7497	0.8263
		(0.000)	(0.000)	(0.000)	(0.000)	(0.412)	(0.413)	(0.000)	(0.290)	(0.262)
	β_2	0.0000	0.0000	0.0000	0.0000	0.4914	0.5238	0.0000	0.7650	0.8308
		(0.000)	(0.000)	(0.000)	(0.000)	(0.423)	(0.425)	(0.000)	(0.297)	(0.264)
PNRFI	β_0	0.0000	0.0000	0.0000	0.0000	0.0000	0.1990	0.0000	0.0000	0.2117
		(0.000)	(0.000)	(0.000)	(0.000)	(0.000)	(0.312)	(0.000)	(0.000)	(0.216)
	β_1	0.0000	0.0000	0.0000	0.0000	0.4939	0.5127	0.0000	0.2761	0.3271
		(0.000)	(0.000)	(0.000)	(0.000)	(0.461)	(0.459)	(0.000)	(0.210)	(0.258)
	β_2	0.0000	0.0000	0.0000	0.0000	0.3318	0.3386	0.0000	0.2373	0.2803
		(0.000)	(0.000)	(0.000)	(0.000)	(0.414)	(0.411)	(0.000)	(0.166)	(0.210)

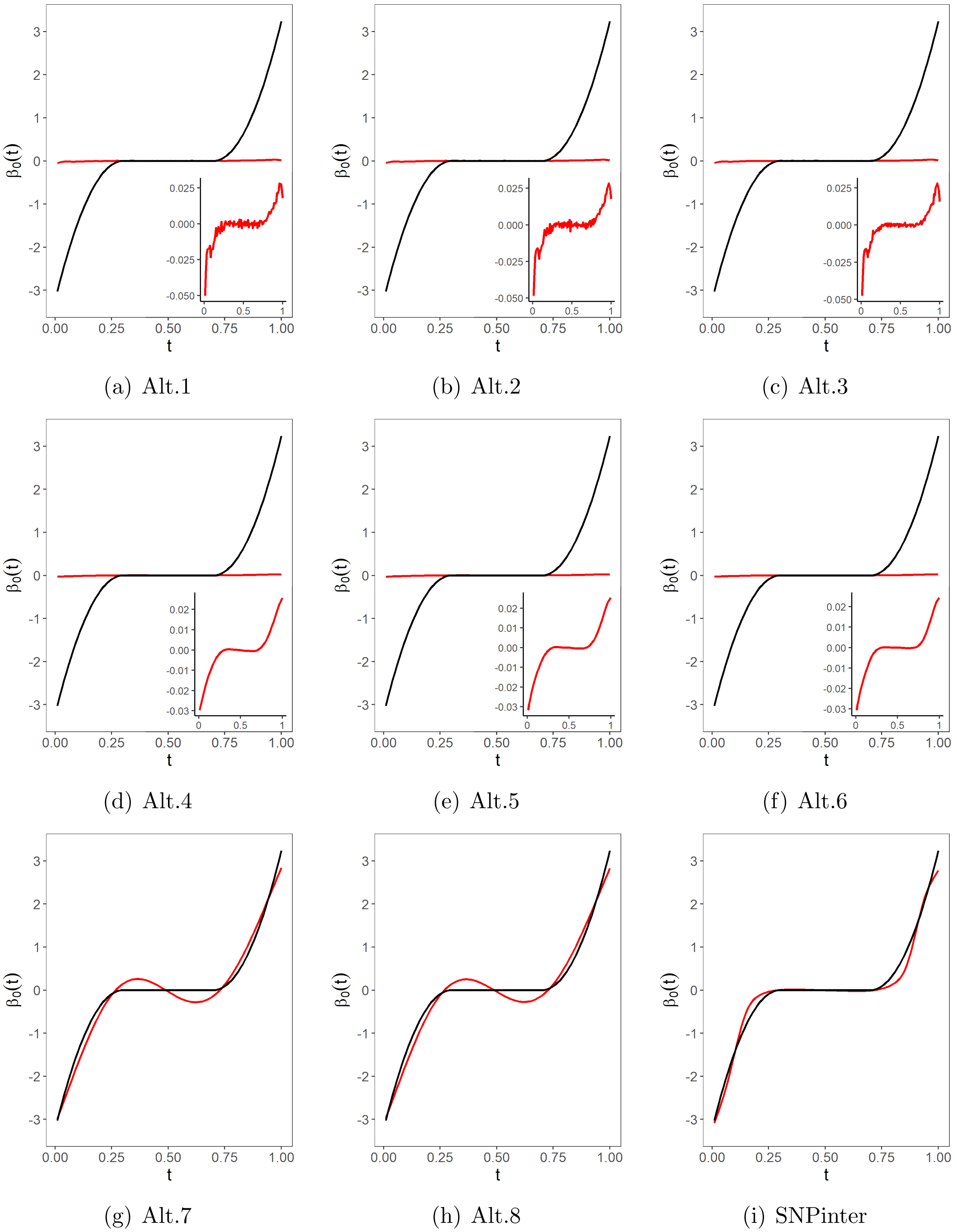

(a) Alt.1 (b) Alt.2 (c) Alt.3

(d) Alt.4 (e) Alt.5 (f) Alt.6

(g) Alt.7 (h) Alt.8 (i) SNPinter

Figure 5: Estimation of $\beta_0(t)$ under Case I and $n = 500$. In each plot, the black line represents the true function $\beta_0(t)$, and the red line represents the mean function of the 500 estimated $\widehat{\beta}_0(t)$.

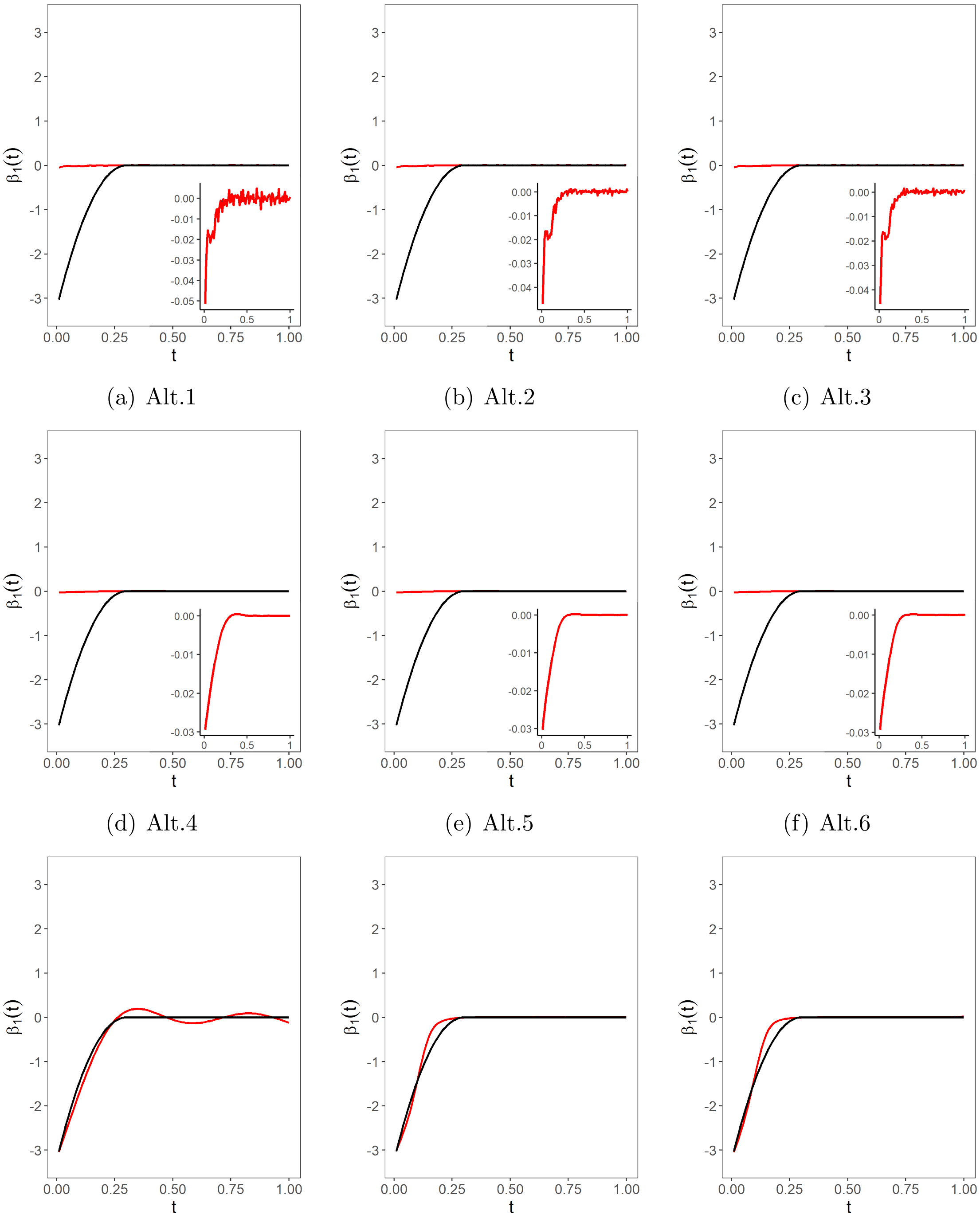

(a) Alt.1 (b) Alt.2 (c) Alt.3

(d) Alt.4 (e) Alt.5 (f) Alt.6

(g) Alt.7 (h) Alt.8 (i) SNPinter

Figure 6: Estimation of $\beta_1(t)$ under Case I and $n = 500$. In each plot, the black line represents the true function $\beta_1(t)$, and the red line represents the mean function of the 500 estimated $\widehat{\beta}_1(t)$.

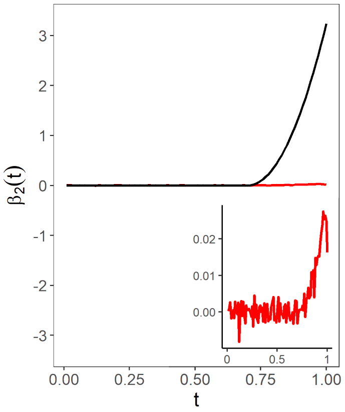

(a) Alt.1

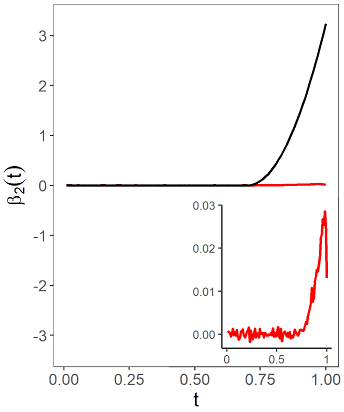

(b) Alt.2

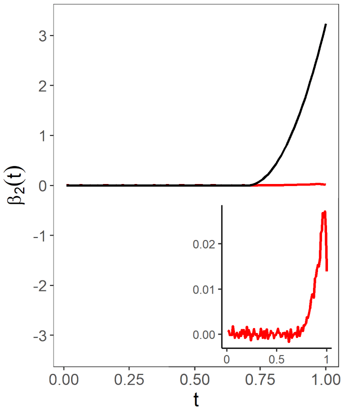

(c) Alt.3

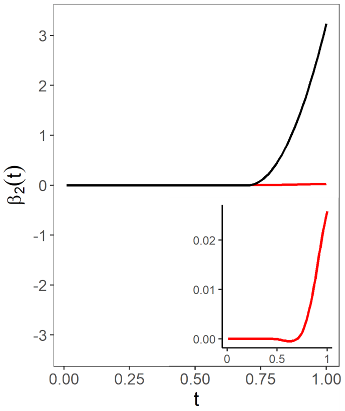

(d) Alt.4

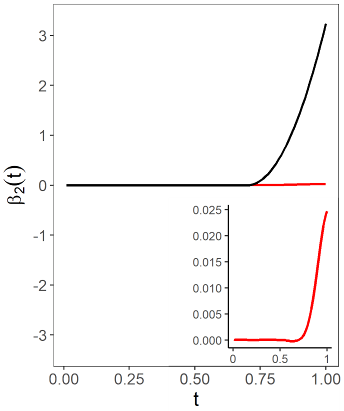

(e) Alt.5

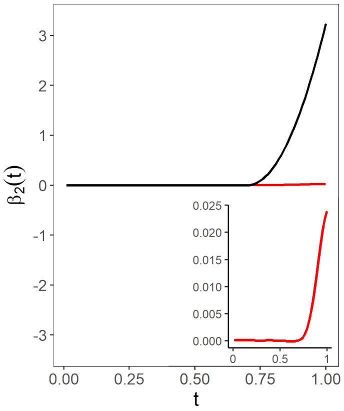

(f) Alt.6

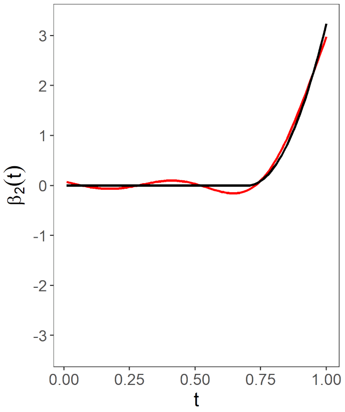

(g) Alt.7

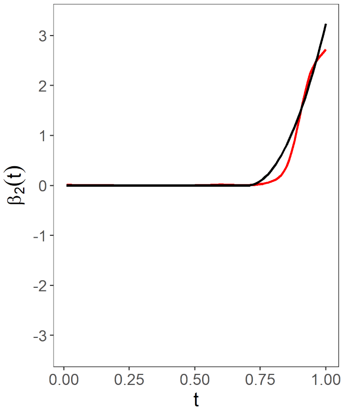

(h) Alt.8

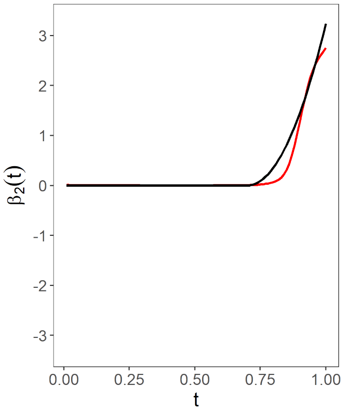

(i) SNPinter

Figure 7: Estimation of $\beta_2(t)$ under Case I and $n = 500$. In each plot, the black line represents the true function $\beta_2(t)$, and the red line represents the mean function of the 500 estimated $\widehat{\beta}_2(t)$.

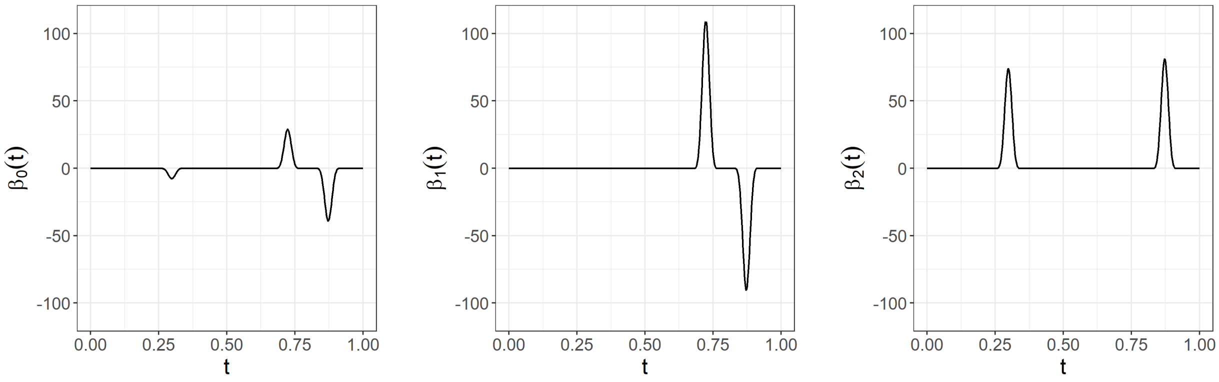

(a) $\beta_0(t)$ (b) $\beta_{\text{gender}}(t)$ (c) $\beta_{\text{age}}(t)$

Figure 8: Estimated effect functions using SNPinter.

The estimation results are summarized in Tables 2 (with different sample sizes) and 4 (with different cases). The selection results are summarized in Tables 3 (with different sample sizes) and 5 (with different cases). It is noted that, to facilitate a direct comparison, the tables have some overlap. Additionally, in Figures 5, 6, 7, we plot the mean estimate versus the true value for $\beta_0(t)$, $\beta_1(t)$, and $\beta_2(t)$, respectively. Overall, the simulation results re-establish the effectiveness of the adopted analysis and "validate" the software functions.

4 Data application

We analyze data from the Childhood Asthma Management Program (CAMP), which consists of 242 children diagnosed with asthma between the ages of 5 and 12. In this analysis, the response variable is FEV1 (forced expiratory volume in 1 second, prebronchodilator), which is a critical biomarker for asthma and has a continuous distribution. For the genetic variants, we consider 21,985 SNPs coded as 0, 1, and 2. We incorporate the interactions between the genetic variant function and two scalar covariates (gender and age) in modeling. We analyze data using the SNPinter approach and function as well as the alternative approaches. The estimation results for the genetic effect function $\beta_0(t)$ and interaction functions (β_{gender}, β_{age}) are shown in Figure 8. It is observed that the estimated effects are locally sparse and respect the "main effect, interaction" hierarchy. For the scalar covariates, the estimated effects are $(\widehat{\alpha}, \widehat{\gamma}_{\text{gender}}, \widehat{\gamma}_{\text{age}}) = (4.5457, -0.0927, -0.2429)$.

To gain some additional insight, we resort to a random splitting based evaluation ap-

Table 6: Average estimates and prediction errors for the intercept and scalar covariate effects based on 100 random partitions. sd in "()".

Method	Estimate			Prediction Error
	$\widehat{\alpha}$	$\widehat{\gamma}_{\text{gender}}$	$\widehat{\gamma}_{\text{age}}$	
Alt.1	4.5352(0.041)	-0.0963(0.034)	-0.2600(0.033)	1.6518(0.300)
Alt.2	4.5465(0.038)	0.0000(0.000)	-0.0286(0.035)	1.0714(0.182)
Alt.3	4.5454(0.037)	0.0000(0.000)	-0.0271(0.037)	1.0688(0.170)
Alt.4	4.5520(0.031)	-0.0976(0.032)	-0.2554(0.035)	0.8525(0.137)
Alt.5	4.5759(0.038)	-0.1467(0.041)	-0.2390(0.036)	0.9408(0.214)
Alt.6	4.5644(0.034)	-0.1186(0.036)	-0.2375(0.035)	0.7790(0.142)
Alt.7	4.6045(0.032)	-0.1218(0.035)	-0.2347(0.034)	0.7721(0.158)
Alt.8	4.5939(0.036)	-0.1189(0.036)	-0.2390(0.032)	0.7758(0.184)
SNPinter	4.5934(0.035)	-0.1149(0.034)	-0.2428(0.035)	0.7508(0.156)

proach. In particular, we randomly split data into a training and a testing set, with a ratio of 4:1. The training data is analyzed using the SNPinter approach/software as well as the alternatives. Prediction is then made for the testing data. To avoid an extreme split, this procedure is repeated 100 times. The average estimates and prediction errors are presented in Table 6. Differences across the approaches are observed. SNPinter has a better prediction performance.

References

Fan, R., Wang, Y., Mills, J. L., Wilson, A. F., Bailey-Wilson, J. E., and Xiong, M. (2013), "Functional linear models for association analysis of quantitative traits," *Genetic epidemiology*, 37(7), 726–742.

Lin, Z., Cao, J., Wang, L., and Wang, H. (2017), "Locally sparse estimator for functional linear regression models," *Journal of Computational and Graphical Statistics*, 26(2), 306–318.

Shi, X., Liu, J., Huang, J., Zhou, Y., Xie, Y., and Ma, S. (2014), "A penalized robust method for identifying gene–environment interactions," *Genetic epidemiology*, 38(3), 220–230.

Wang, J.-L., Chiou, J.-M., and Müller, H.-G. (2016), "Functional data analysis," *Annual Review of Statistics and Its Application*, 3, 257–295.

Wu, M., Zhang, Q., and Ma, S. (2020), "Structured gene-environment interaction analysis," *Biometrics*, 76(1), 23–35.

Zhang, C.-H. (2010), "Nearly unbiased variable selection under minimax concave penalty," *The Annals of statistics*, 38(2), 894–942.
